# BDNF VAL66MET MODERATES THE EFFECTS OF HYPERTENSION ON EXECUTIVE FUNCTIONING IN OLDER ADULTS DIAGNOSED WITH AMCI

**DOI:** 10.1093/geroni/igac059.1727

**Published:** 2022-12-20

**Authors:** Peter Louras, Lisa Brown, Rowena Gomez, Stacie Warren, Kaci Fairchild

**Affiliations:** VA Palo Alto Health Care System, Palo Alto, California, United States; Palo Alto University, Palo Alto, California, United States; Palo Alto University, Palo Alto, California, United States; Palo Alto University, Palo Alto, California, United States; VA Palo Alto Healthcare System, Palo Alto, California, United States

## Abstract

Brain-derived neurotrophic factor (BDNF) has a demonstrated role in promoting memory functions and neuronal survival. A common variant in the BDNF gene, the Val66Met polymorphism, has been found to reduce BDNF expression and increase vulnerability to neurocognitive impairments and age-related cognitive declines. Research suggests that BDNF Val66Met may be associated with hypertension, a well-established risk factor for health and brain functioning across the lifespan. While BDNF is most studied for its effect on learning and memory, its role in moderating other cognitive domains is not as well understood, especially those most affected by hypertension. Furthermore, no study to date has investigated these relationships exclusively with older adults and those at increased risk for cognitive decline. Therefore, the aim of this study was to investigate the effect of BDNF Val66Met and hypertension on the executive function ability of older adults (mean age 71.3±9.2 years) diagnosed with amnestic Mild Cognitive Impairment (aMCI) (N = 108). Results showed that BDNF Val66Met moderated the relationship between hypertension and executive functioning, such that hypertensive carriers of the BDNF Met allele performed significantly worse compared to Val-Val homozygotes, and independent to the effects of aging. These results indicate that genetic and vascular risk interactions can predispose older adults with aMCI to impairments in multiple cognitive domains, and potentially increase susceptibility to further declines or conversion to dementia. These findings have implications for future research on dementia disease pathology, and highlight the importance of strategies that target fixed and modifiable risk factors to promote cognitive resilience.

